# The value of magnetic resonance imaging as a biomarker for amyotrophic lateral sclerosis: a systematic review

**DOI:** 10.1186/s12883-016-0672-6

**Published:** 2016-08-27

**Authors:** G. Grolez, C. Moreau, V. Danel-Brunaud, C. Delmaire, R. Lopes, P. F. Pradat, M. M. El Mendili, L. Defebvre, D. Devos

**Affiliations:** 1 Department of Movement Disorders and Neurology, Lille University Hospital, Faculty of Medicine, University of Lille, Lille, France; 2 INSERM U1171, Lille University Hospital, Faculty of Medicine, University of Lille, Lille, France; 3 Department of Neuroradiology, Lille University Hospital, Faculty of Medicine, University of Lille, Lille, France; 4 Laboratoire d’Imagerie Biomédicale, Sorbonne Universités, UPMC Univ Paris 06, CNRS, INSERM, Paris, France; 5 Département des Maladies du Système Nerveux, Groupe Hospitalier Pitié-Salpêtrière, APHP, Paris, France; 6 Department of Medical Pharmacology, Lille University Hospital, Faculty of Medicine, University of Lille, Lille, France

**Keywords:** Amyotrophic lateral sclerosis, Magnetic resonance imaging, Morphometry, Diffusion tensor imaging, Magnetic resonance spectroscopy, Spinal cord, Biomarkers

## Abstract

**Background:**

Amyotrophic lateral sclerosis (ALS) is a fatal, rapidly progressive neurodegenerative disease that mainly affects the motor system. A number of potentially neuroprotective and neurorestorative disease-modifying drugs are currently in clinical development. At present, the evaluation of a drug’s clinical efficacy in ALS is based on the ALS Functional Rating Scale Revised, motor tests and survival. However, these endpoints are general, variable and late-stage measures of the ALS disease process and thus require the long-term assessment of large cohorts. Hence, there is a need for more sensitive radiological biomarkers. Various sequences for magnetic resonance imaging (MRI) of the brain and spinal cord have may have value as surrogate biomarkers for use in future clinical trials. Here, we review the MRI findings in ALS, their clinical correlations, and their limitations and potential role as biomarkers.

**Methods:**

The PubMed database was screened to identify studies using MRI in ALS. We included general MRI studies with a control group and an ALS group and longitudinal studies even if a control group was lacking.

**Results:**

A total of 116 studies were analysed with MRI data and clinical correlations. The most disease-sensitive MRI patterns are in motor regions but the brain is more broadly affected.

**Conclusion:**

Despite the existing MRI biomarkers, there is a need for large cohorts with long term MRI and clinical follow-up. MRI assessment could be improved by standardized MRI protocols with multicentre studies.

## Background

Amyotrophic lateral sclerosis (ALS) is a neurodegenerative disease that mainly affects the motor system. At present, the only drug to have produced an increase in patient survival in a controlled clinical trial is riluzole [1]. The condition is always fatal; the median survival time after onset is 36 [2], although there are great individual variations [3]. Although ALS is a clinically recognizable condition (in terms of the pattern of progressive upper and lower motor neuron degeneration), the clinical presentation and progression are heterogeneous [4]. Moreover, there are now strong reasons for considering a broader aetiological and pathogenic spectrum [5]. This clinical heterogeneity is observed in other neurodegenerative diseases, such as Parkinson’s disease (PD). Nevertheless, it has been found that all PD patients display degeneration and iron overload of the substantia nigra, which might become a surrogate biomarker [6]. In ALS, iron overload in the motor cortex was confirmed [7] after being suspected in mouse models [8]. Ongoing drug trials in the field of neurodegenerative disease are mainly seeking to establish neuroprotection and neurorestoration. Since neuroprotection can never be unambiguously demonstrated in a given patient, the concept of disease-modifying drugs (with a slowing of the disease progression) has arisen. At present, the evaluation of a drug’s clinical efficacy in ALS is based on the ALS Functional Rating Scale Revised, motor tests, survival or a combination of these measures (such as the Combined Assessment of Function and Survival (CAFS)) [9, 10]. However, these endpoints are general, variable and late-stage measures of the ALS disease process and thus require the long-term assessment of large cohorts. These assessments are risky and expensive, which considerably limits the number of trials being conducted. A sensitive surrogate biomarker might help to (i) reduce the sample size in pilot studies, (ii) better define the sample size required in Phase III clinical trials (by comparison with clinical scales) and (iii) define endophenotypes for separate assessment in clinical trials.

Biomarkers are typically divided into “wet biomarkers” and “dry biomarkers”. Wet biomarkers are biological substances that are measured in a body fluid (such as whole blood, serum, plasma, saliva or cerebrospinal fluid (CSF)). “Dry biomarkers” are based on functional scales, task performance, electrophysiology or imaging. Magnetic resonance imaging is a widely available and non-invasive technique, which makes it an appropriate candidate for biomarkers. Although early reports stated that ALS starts in the spinal cord, the initial site of neurodegeneration has not been unambiguously identified; disease may start in the spinal cord, in the motor cortex or at both sites simultaneously [11, 12]. The development of radiological biomarkers in ALS is facilitated by the hypothesis that the pathological changes in ALS radiate out from the initial spinal and brain areas in which the most severe upper and lower motor neuron dysfunction respectively occur (from symptom onset onwards) with variability in the predominance of upper and lower motor neuron dysfunction [13]. The neurodegeneration then progresses along the motor neurons of the ventral horn of the spinal cord and into the corresponding areas of the motor cortex [13]. Despite this modelization, the pattern of onset and progression remains complex [13]. Hence, MRI of the spinal cord should be an early, sensitive marker of subtle degeneration-related changes. However, MRI of the spinal cord is complicated by a number of technical difficulties (relative to MRI of the brain). Firstly, the magnetic field is inhomogeneous because the spinal cord is close to bone, soft tissues and air. Secondly, the spine has a small cross-sectional dimension, which thus requires high-resolution data acquisition. Thirdly, the spine is subject to motion of the CSF [14] and physiological motion due to breathing [15].

Many different MRI techniques and sequences – including voxel-based morphometry (VBM), diffusion tensor imaging (DTI), magnetic resonance spectroscopy (MRS), iron-sensitive sequences (T2*, R2* and SWI) and functional MRI (fMRI) - are available for studying ALS-related changes in the brain or spinal cord. Hence, we reviewed the correlations between MRI findings and clinical scores in ALS, and sought to establish whether particular sequences may have value as surrogate biomarkers in this disease.

## Methods

We searched the PubMed database with the following keywords: “ALS AND MRI OR amyotrophic lateral sclerosis AND magnetic resonance imaging”, “ALS AND VBM OR amyotrophic lateral sclerosis AND magnetic resonance imaging ”, “ALS AND MR spectroscopy OR amyotrophic lateral sclerosis AND magnetic resonance spectroscopy”, “amyotrophic lateral sclerosis AND diffusion tensor imaging OR ALS AND DTI”, “amyotrophic lateral sclerosis AND functional magnetic resonance imaging OR ALS AND fMRI”. There was no limitation on the publication date but only publications in French or English were considered. We included (i) general MRI studies with a control group and an ALS group and (ii) longitudinal follow-up studies (even if a control group was lacking). We excluded therapeutic trials articles, reviews, studies focused only on particular subgroups of ALS patients such as cognitive impairment, genetic mutations.

## Results

A total of 1336 articles were identified using database searching, 651 were recorded after duplicates removal. 405 were excluded (not ALS, not MRI or, therapeutic clinical trials, not English or French, reviews) and 130 articles were finally excluded (studies focused only on particulars subgroups of patients, other MRI sequences or lacking of a control group). We analysed 116 articles.

### Brain imaging

#### Structural magnetic resonance imaging (Table 1)

**Table 1:** The main results of volumetric studies

Publication	Numbers of patients/controls	Main results	Main clinical correlations
Abdulla et al., [39, 80]	58/29	Low volume in the right hippocampus	Verbal memory test performance is correlated with the left hippocampal volume.
Agosta et al., [21]	25/18	GM volume reduction in right precentral, left inferior frontal cortex and temporal superior cortex.	Negative with ALSFRS-R and DD.
Agosta et al., [35]	44/26	Reduction in cortical thickness in the precentral, frontal, limbic, parietal, temporal and occipital lobes.	DPR is correlated with the mean cortical thickeness of left sensorimotor cortex.
Bede et al., [37]	39/44	GM volume reduction in the thalamus, caudate nucleus, hippocampus, left putamen.	Not available.
Canu et al., [23]	23/24	GM volume reduction in the precentral, right opercular and angular cortex, WM volume reduction in the frontal lobe (especially the subcortical motor areas) and the temporal lobe.	Not available.
Cerami et al., [36]	14/20	Low GM density in the anterior cingulate and right inferior frontal gyri.	Not available.
Chang et al., [22]	20 (10 ALS and 10 ALS-FTD)/22	GM volume reduction in the precentral, frontal and temporal cortex and left posterior thalamus.	Not available.
Chapman et al., [40]	25/22	No intergroup difference in the corpus callosum area	Not available.
Devine et al., [27]	30/17	GM volume reduction in the left precentral cortex, left frontal gyrus, medial frontal gyri and anterior cingulate gyri.	Increased GM volume reduction in the dominant (left) motor cortex irrespective to the side of limb onset. Asymmetric GM reduction of the left somatosensory cortex and the temporal gyri in in right limb onset ALS
Ellis et al., [26]	16 (8 bulbar onset and 8 limb onset)/8	GM volume reduction in the superior, middle and medial frontal cortex. WM volume reduction in the right frontal cortex.	Compared to limb onset ALS, bulbar onset ALS showed GM volume reduction in the brainstem, the cerebellum and in the fusiform gyri (BA 34) and WM volume reduction along the left corticospinal tract.
Grosskreutz et al., [18]	17/17	GM volume reduction in the precentral, frontal and parietal cortex. No WM atrophy.	ALSFRS-R correlated positively with GM volume reduction in the right medial frontal gyrus (BA 10).
Kassubek et al., [32]	22/22	GM volume reduction in the right primary motor cortex and the left medial gyrus and bilateral inferior temporal gyrus.	Negative with ALSFRS-R and DD.
Meadcroft et al., 2014	8/6	Post mortem study; loss of signal difference between WM and GM (mainly in the primary motor cortex).	Not available.
Mezzapesa et al., [19]	16/9	GM volume reduction in the precentral, frontal and parietal cortex.	Not available.
Mezzapesa et al., [20]	29/20	GM volume reduction in the precentral, frontal, right temporal and right occipital cortex.	Different patterns of volume reduction in ALS group, high UMN burden, spinal onset and faster progression compared to controls.
Sach et al., [33]	15/12	No difference -study not designed to voxel-based morphometry.	Not available.
Sage et al., [34]	28/26	No difference -study not designed to voxel-based morphometry.	Not available.
Schuster et al., [25]	93 (60 ALS, 17 with dominant UMN, 16 with dominant LMN)/67	Correlations between the site of onset and upper vs. lower motor neuron involvement, with focal reductions in cortical thickness in the precentral cortex.	Different patterns of volume reduction in ALS subgroups: bulbar and spinal UMN signs, only spinal UMN signs, classical ALS, UMN ALS-variants, LMN ALS-variants or sites of onset compared to controls. GM reduction is most important in the left precentral cortex in bulbar onset vs. limb onset. No correlations of MRI with DPR, DD and ALSFRS-R.
Thivard et al., [24]	15/25	GM volume reduction in the precentral, frontal and temporal cortex (especially the hippocampus), the parietal and occipital cortex, the thalamus and the cerebellum. No difference in WM.	Not available.
Verstraete et al., [30]	12/12	GM reduction in the right and left precentral cortex.	Not available.
Walhout et al., [29]	153 (112 ALS, 19 UMN, 19 LMN)/60	GM reduction in the right and (predominantly) left precentral cortex and the right paracentral cortex.	Bulbar and arms ALSFRS-R subscores are correlated with cortical thickness of corresponding precentral cortex areas of the motor homunculus. DPR is correlated with the thickness of the right inferior temporal cortex, the postcentral cortex and the right paracentral cortex.
Westenberg et al., 2014	112/60	GM volume reduction in the hippocampus and left subiculum.	Larger ventricles are correlated to a lower ALSFRS-R score. Smaller basal ganglia, smaller limbic structures and larger ventricles are associated with shorter survival.
Zhang et al., [28]	43/43	GM reduction in the left precentral cortex, left supplemental area and left postcentral gyrus. Reduction in neocortex volume, GM and WM volume and brain parenchymal fraction.	GM volume reduction is most important in the motor cortex of the contralateral hemisphere of the limb of onset. ALSFRS is positively correlated with GM density in the left postcentral cortex and DPR is negatively correlated with GM density in the right precentral cortex
Zhu et al., [31, 87]	22/22	GM reduction in the right and left precentral cortex.	Negative with DD, DPR and all other clinical parameters.

*GM* grey matter, *WM* white matter, *BA* Brodman area, *DD* disease duration, *DPR* Disease progression rate, *UMN* upper motor neuron

Three methods based on a three-dimensional, T1-weighted sequence have been applied to ALS: VBM, volume analysis and the measurement of cortical thickness. As a volume-based analysis, VBM involves the automatic segmentation of grey matter and inter-subject comparison of the local grey matter density [16, 17]. After all the data have been transformed in the same stereotaxic space, the images are partitioned and corrected for the separation between white matter, grey matter and CSF as a function of voxel intensity [17]. Measurement of cortical thickness is a surface-based technique analysis in which the white matter and pial surfaces are automatically extracted by applying image analysis packages such as Freesurfer (http://surfer.nmr.mgh.harvard.edu/).

Most of these techniques have evidenced atrophy in the precentral gyri [18–32]. However, some DTI studies used VBM to show that ALS vs. control differences in the fractional anisotropy (FA) of the precentral gyri were not due to atrophy [33, 34]. In other studies, cortical atrophy was not limited to the precentral gyri but extended to other regions of the frontal lobe [18–24, 26–28, 32, 35] - especially in apathetic patients with involvement of the cingulate cortex [27, 36]. Other regions involved include several parts of the temporal cortex [19–24, 32, 35], the hippocampus [24, 37–39], the parietal cortex (mostly in the post-central cortex [18, 21, 23, 28]) and the insula [24]. Occipital grey matter atrophy [19, 20, 24, 35] and cerebellar atrophy were less common [24]. When considering the basal ganglia, a low volume of the thalamus [22, 24, 37] and the caudate nucleus [37] have been observed. Few studies have evidenced a relative reduction in the volume of white matter (mainly in the frontal and temporal lobes) [23]. There were no marked differences in the corpus callosum [40]. Whole-brain analyses have evidenced low volumes in the neocortex, grey matter, white matter [28] and brain parenchyma [28, 32]. A longitudinal study showed (i) a volume reduction in the left subiculum, the right hippocampal area CA4 and the right dentatus gyrus and (ii) an enlargement of the ventricles in 39 of the 112 ALS patients [38].

##### Clinical correlations

Specific MRI abnormalities have been correlated with clinical scores, the disease stage (as estimated with the original or revised ALS Functional Rating Scale (ALSFRS and ALSFRS-R, respectively), the speed of disease progression (i.e. the change in ALSFRS or ALSFRS-R score, divided by the disease duration or the time interval between two clinical assessments), the disease duration and the survival time. Lower ALSFRS or ALSFRS-R scores were associated with larger ventricles and greater volume loss in the basal ganglia [38], and volume changes in several parts of the frontal lobe (such as Brodman area 10) [18]. In another study, a higher ALSFRS-R progression rate was associated with MRI changes in the left sensorimotor area; cortical thickness was lower in patients with predominantly upper motor neuron impairment (especially in the precentral gyrus and the left paracentral lobule), whereas patients with predominantly lower motor neuron impairment showed less difference relative to controls [20]. Grey matter density was lower in the cingulate and right inferior frontal gyrus in patients with impaired emotional empathy [36]. Poor survival in ALS is correlated with volume loss in the basal ganglia and limbic structures [38]. The initial side of limb weakness in ALS patients was not correlated with contralateral brain cortical loss of volume [27]. Although atrophy was predominant in the left sensorimotor cortex [27, 28], it was not related to the side of onset [27]. A subgroup analysis (by side of onset) found more widespread atrophy in the contralateral brain cortex than in the group of ALS patients as a whole [28]. Precentral cortical thickness was lower in patients with upper motor neuron clinical features than in those with lower motor neuron clinical features and in the ALS group as a whole [29].

In a study with a post mortem T1 sequence and histological assessment, an abnormally small difference in intensity between cortical grey matter and subcortical white matter was observed not only in the motor cortex (including the precentral gyrus, supplemental motor area and premotor cortex) but also in somatosensory areas and the primary visual cortex. These signal abnormalities were linked to neuron loss and an elevated number of astrocytes [41].

#### Diffusion tensor imaging (Table 2)

**Table 2:** The main results of DTI studies

Publication	Numbers of patients/controls	Fractional anisotropy	Mean diffusivity or apparent diffusion coefficient	Radial and axial diffusivity	Main clinical correlations
Abe et al., [64]	7/11	Low in parts of the right frontal lobe and in the left subcortical precentral area			Not available
Agosta et al., [21]	25/18	Low in parts of CSTs, the CC, parts of the frontal lobe and parts of the temporal lobe	No difference		Negative
Bede et al., [75]	27/42	Low along CSTs, the CC, cerebellum, brainstem, occipital lobe and opercular and insular regions		RD was low in the cerebellum, brainstem, occipital lobe and opercular and insular regions	Not available
Canu et al., [23]	23/14	Low in subcortical parts of CSTs and other parts of the frontal lobe	Elevated in parts of the CSTs, left postcentral gyrus, left insula, parts of the left temporal lobe, right angular cortex, parts of the frontal lobe, CC, parts of the occipital lobe and parts of the cerebellum		Correlation between MD in bilateral orbitofrontal region and DD
Chapman et al., [67]	21/21	Low in the CC, corona radiata and PLIC		RD was low in the CC, corona radiate and PLIC. AD was elevated in the left corona radiata and the internal and external capsules	Correlation between FA in parts of CC and ALSFRS-R and negative correlation with DD, correlation between RD in parts of CC and DD
Ciccarelli et al., [45]	26/41	Low along CSTs, the CC, anterior limb of the internal capsule, external capsule, parts of frontal lobe WM and postcentral gyri			Correlation between DPR and FA in left cerebral peduncle, right PLIC, right corona radiate right WM adjascent to the precentral gyrus and CC
Ding et al., [74]	10/10		Elevated in the PLIC		Not available
Ellis et al., [51]	22/20	Low along CSTs	Elevated along CSTs		Correlation between mean diffusivity in CST and DD and between FA along CSTs and ALS severity scale and spasticity scales.
Filippini et al., [56]	24/24	Low along CSTs and in the CC		Elevated RD in WM linked to the primary motor and premotor cortex and the CC	Inverse correlation between FA and UMN score along CSTs, correlation between FA and ALSFRS-R and DD along CSTs
Foerster et al., [55]	29/30	Low along CSTs			Correlation between FA and ALSFRS-R along CSTs
Iwata et al., [49]	31/31	Low along CSTs			Negative
Iwata et al., [50]	18/19	Low along CSTs and in the motor part of the CC	Elevated along CSTs		Correlation between DD and FA along CSTs. Inverse correlation between FA in CSTs and global and localized UMN impairment score and between FA along CST and UMN rapidity index.
Kassubek et al., [12, 73]	111/74	Low along CSTs			Not available
Keil et al., [61]	24/24	Low in parts of CSTs, supplementary motor area, CC, parts of the frontal lobe and the parahippocampal area	Elevated in motor areas, parts of CSTs, parts of the frontal and temporal lobe, and the postcentral gyrus		Correlation between FA along CSTs and ALSFRS-R. Correlation between executives functions (sfs36 score) and FA in cerebellum. At 6 months, negative correlation between FA along CSTs and DD correlation between FA along CSTs and frontal WM and ALSFRS-R. Correlation between FA in CST at brainstem level and executives functions, negative correlation between ADC in the cerebellum and parahippocampal gyri and executives functions (sfs36)
Keller et al., [68]	33/30	Low in the corona radiata and CC			Negative
Liu et al., 2014	19/13	Low along CSTs			Correlation between FA along left CST and ALSFRS-R
Menke et al., [77]	21/0 (follow-up study)	Progressive reduction in the PLIC			Correlation between FA at baseline and DPR
Metwalli et al., [69]	12/19	Low along CSTs, the CC	Elevated along CSTs, the CC	AD and RD were elevated along CSTs, RD was elevated in the CC, parts of frontal and parietal lobe	Negative
Muller et al., 2011	19/19	Low in parts of CSTs, the parahippocampal area, insula and brainstem			Correlation between FA in parts of CSTs and ALSFRS-R
Nickerson et al., [46]	2/0 (follow-up study)	A linear reduction along CSTs during one year follow-up			Not available
Poujois et al., [65]	19/21	Low in CSTs from the left corona radiate to the precentral gyrus and in both cerebral peduncles			Muscular strength is lower on the right side corresponding to the lower FA in the left CST
Prell et al., [60]	17/17	Low in parts of CSTs and the cingulate gyrus	Elevated parts of CSTs, parts of frontal lobe, cingulate gyrus, parahippocampal region, CC, cerebellum.		Correlation between FA in internal capsule and contralateral strength of the lower limb. Different patterns in FA and ADC of bulbar and limb onset compared to controls.
Prudlo et al., [70]	22/21	Low throughout the CSTs, the anterior limb of the internal capsule, thalamic radiations, the CC, association fibres and the middle cerebellar peduncle			Correlation between FA in many voxels of a whole DTI brain analyses with ALSFRS-R
Pyra et al., [59]	14/14	Low in left precentral gyrus			Correlation between spasticity and ADC in contralateral precentral gyrus
Rajagopalan et al., [63]	47/10	Low in the left subcortical motor area and right PLIC		AD was low in the PLIC. RD was elevated in the PLIC of patients with T2 hyperintensities in the CSTs	Not available
Rosskopf et al., [57]	100/93	Low along CSTs			Correlation between FA along CSTs and ALSFRS-R
Sach et al., [33]	15/12	Low in parts of CSTs, premotor areas, CC and right thalamus			Low FA in patients without UMN signs in parts of CST, CC and right thalamus. No correlation available with clinical score
Sage et al., [34]	28/26	Low along CSTs and the right postcentral gyrus	Elevated in parts of CSTs		Correlation between FA and ALSFRS in several parts of CSTs and in prefrontal lobe
Sage et al., [52]	28/26	Low in parts of CSTs, parts of the frontal lobe, insula, hippocampus, cerebral peduncles and CC	Elevated along CSTs, hippocampus, insula, parts of the temporal and frontal lobe and CC		Correlation between FA along CSTs, in prefrontal area and ALSFRS. Negative correlation between MD along CST, hippocampus, cerebellum, parietal and temporal lobe and ALSFRS.
Sarica et al., [53]	14/14	Low in right CSTs and left anterior thalamic radiations	Elevated in right CSTs, cingulum and left anterior thalamic radiations	RD elevated in right CSTs and left anterior thalamic radiations. AD elevated in the right cingulum	Negative (p ≤ 0.05)
Schirimrigt et al., 2007	10/20	Low along CSTs (nb: study with a technical objective)			Correlation between FA along CSTs and DD
Stagg et al., [47]	13/14	Low along CSTs	Elevated along CSTs		Negative
Tang et al., [58]	69/23	Low along CSTs, in frontal WM and the genu of the CC	Elevated in the centrum semi-ovale and frontal and parietal WM		Not available
Thivard et al., [24]	15/25	Low along CSTs, premotor cortex, right thalamus, insula, parts of parietal lobe	Elevated in the motor cortex, premotor cortex, insula, hippocampus, and right superior temporal gyrus		Correlation between FA along CST, insula, premotor cortex, cingulum, precuneus, CC and ALSFRS-R, negative correlation between FA in CC and centrum semiovale and DD
Verstraete et al., [30]	12/12	Low in the rostral part of CSTs and the CC			Not available
Wang et al., [71]	16/17	Low CST volume in DTI			Negative
Yin et al., [48]	8/12	Low along CSTs			Not available
Zhang et al., [62]	17/19	Low in parts of CSTs	Elevated in parts of the CSTs		Correlation between FA in right superior CST and ALSFRS-R and motor subscore of ALSFRS-R

*FA* fractional anisotropy, *MD* mean diffusivity, *ADC* apparent diffusion coefficient, *RD* radial diffusivity, *AD* axial diffusivity, *CST* corticospinal tract, *PLIC* posterior limb of the internal capsule, *CC* corpus callosum, *DD* disease duration, *DPR* disease progression rate, *UMN* upper motor neuron, *LMN* lower motor neuron

DTI (also known as magnetic resonance tractography) is based on the random diffusion (Brownian motion) of molecules. In a spherical volume, the diffusion of water has no main direction and its diffusion in the three directions (λ1, λ2 and λ3) is equally likely. However, this is no longer the case in an ellipsoid or cylindrical volume and the diffusion is anisotropic. λ1 is the volume’s main axis (axial diffusivity, AD) and λ2 and λ3 are the minor axes (radial diffusivity, RD). By analysing the diffusion of water in three directions, four parameters can be defined: FA, RD, AD and mean diffusivity (MD, which is the average of diffusion in the λ1, λ2 and λ3 axis, also referred to as the apparent diffusion coefficient (ADC)). The first three parameters describe the spatial variation of water movement and are related to the orientation of the studied structures. In contrast, MD corresponds to the mean displacement of the water molecules within the volume. The main application of DTI is the analysis of the anatomical bundles that compose the white matter of the brain and the spinal cord [42–44]. Whereas DTI corresponds to the analysis of the above-mentioned parameters in a defined volume, tractography corresponds to the analysis of a complete white matter tract.

##### Fractional anisotropy in ALS

Low FA along entire corticospinal tracts (CSTs) is the most common observation in patients with ALS [34, 45–58]. However, some studies have only reported low FA in one or several parts of a CST: the subcortical white matter of the precentral gyrus [23, 24, 30, 33, 59–65], the corona radiata [24, 60–62, 66–68], the posterior limb of the internal capsule (PLIC) [24, 33, 60, 62, 63], the cerebral peduncles [21, 59, 61] and the pons [61]. Lastly, only one study failed to observe a difference in FA along a CST in ALS patients by region of interest approach whereas tract based spatial statistics (TBSS) showed decreased FA in corona radiate and corpus callosum [68].

Low FA has also been observed in other regions: the frontal lobe (excluding the CST) [21, 24, 33, 34, 45, 52, 56, 57, 61, 64], the cingulum [60], the corpus callosum [21, 33, 45, 50, 52, 58, 61, 67–69], the parietal lobe [24, 34, 45], the temporal lobe [21], parahippocampal areas [61], the hippocampus [34], the insula [34, 52], the cerebellum [61] and the thalamus [24, 33, 53]. In a whole-brain comparison, many other small areas of ALS-modified white matter tracts were observed [70].

A tractography study revealed that the CST volumes of ALS patients are lower than in controls [71]. A DTI study using a tract of interest-based fibre tracking found that the radiological results mirrored the neuropathological staging system [72], suggesting that the disease process extended in the following sequence: the corticospinal tract (stage 1), the corticorubral and corticopontine tracts (stage 2), the corticostriatal tract (stage 3) and the proximal portion of the perforant pathway (stage 4) [73]. The researchers suggested that this MRI-based staging system could be used as a surrogate marker of disease progression in clinical studies of ALS.

##### Diffusivity in ALS

The most common observation in patients with ALS is a relative increase in MD, reflecting changes in white matter tracts. The MD was elevated along entire CSTs [51, 53, 69] and in parts of CSTs, including subcortical white matter in the precentral gyrus [23, 34, 58, 60, 61], the corona radiata [34, 60], the PLIC [23, 34, 60, 74], the cerebral peduncles and the pons [34]. Diffusivity was also elevated in several parts of the frontal lobe [23, 24, 52, 58, 60, 61], the temporal lobe [23, 24, 52, 61], the hippocampal region [24, 34, 52], the parahippocampal region [60], the parietal lobe [23, 58, 61], the insula [23, 24, 52], the cingulum [53, 60], the occipital lobe [23], the cerebellum [23, 60] (especially in C9ORF72-positive patients) [75], the corpus callosum [23, 52, 60, 69] and the thalamus [53]. Conversely, two studies did not detect any differences in diffusivity in ALS patients vs. controls [21, 63].

##### Radial and axial diffusivity in ALS

Several studies have found elevated RD and AD values in the right CST, the right cingulum and the left anterior thalamic radiations (on the basis of changes in FA or MD) [53]. In the PLIC, AD is low [63] and RD is elevated [67] - especially in patients presenting with T2 hyperintensities in CSTs [63]. RD is elevated in white matter connected to the primary motor cortex, the premotor cortex [56] and the corpus callosum [56, 67, 69]. Lastly, RD and AD are elevated in the cerebellum of C9ORF72-positive patients [75].

##### Longitudinal studies

In longitudinal analyses, low FA is observed in the right superior CST [62], precentral subcortical regions, mesencephalic CSTs and parts of cerebellum [61]. FA was found to fall in a progressive, linear manner along the CSTs [34, 46, 76], and the diffusivity of the external and internal capsule was elevated [61]. AD is elevated in CSTs [77]. However, a recent longitudinal study found that white matter defects (as assessed by DTI) progressed much slowly that in the grey matter defects - notably in the basal ganglia [78].

##### Clinical correlations

Many studies have found that disease progression (as defined by the change over time in the ALSFRS or ALSFRS-R score) is correlated with the changes in FA in CSTs [24, 34, 45, 61, 62, 77, 79]. Furthermore, the disease duration is correlated with FA in entire CSTs or parts of CSTs [24, 50, 54, 56, 61, 77]. Conversely, the disease duration was negatively correlated with FA in the cerebellum [61], the subcortical white matter of insula, the ventrolateral premotor cortex, the cingulum, the precuneus and the splenium of the corpus callosum [24]. Lower-limb muscle strength was correlated with the contralateral FA in CSTs [60]. Diffusivity values in CSTs and in the parietal lobe, temporal lobe and cerebellum were also correlated with disease progression [52, 79]. Diffusivity in CSTs was correlated with disease duration [79], and contralateral diffusivity in CSTs was correlated with spasticity [59].

Whereas both limb-onset patients and bulbar-onset patients differed from controls in terms of FA along CSTs, the two patient groups had similar values [60]. The CSTs were more impaired in bulbar-onset patients than in limb-onset patients when considering FA, MD and RD but not AD [80]. Tractography revealed a large number of differences between controls and patients defined as definite/probable according to the El Escorial criteria, whereas there were fewer differences between controls and a possible/suspected group [79].

##### Neurophysiological correlations

FA in CSTs was related to the central conduction time in a transcranial magnetic stimulation study [33, 49] (Iwata et al., [49]; Sach et al., [33]).

##### Diagnostic accuracy

An individual patient data meta-analysis of CST studies concluded that DTI lacks sufficient diagnostic discrimination [81]. The pooled sensitivity and specificity were only 0.68 and 0.73, respectively. Researchers have also suggested that a multimodal approach (combining DTI with methods such as MRS) may be more promising [55].

#### Magnetic resonance spectroscopy (Table 3)

**Table 3:** main results of magnetic resonance spectroscopy studies

Publication	Numbers of patients/controls	Main results	Main clinical correlations
Bowen et al., [94]	18/12	Cho and ins were elevated in the MC. NAA and Cr were correlated in left MC	Correlation between ins in MC and UMN disability, negative correlation between Naa in MC and UMN disability, higher Cho in sever UMN disability group
Cervo et al., [90]	84/28	NAA/(Cho + Cr) was low in the MC	Negative
Foerster et al., [95]	10/9	Low gamma aminobutyric acid in the MC but not in WM	Negative
Foerster et al., [55]	29/30	NAA and gamma aminobutyric acid were low and ins was elevated in the left MC	Negative correlation between gamma aminobutyric acid in MC and DD, correlation between Naa peak in MC and ALSFRS-R
Govind et al., [92]	38/70	NAA was low and Cho was elevated in most parts of CST, and Cho/NAA was elevated in all parts of the CSTs	Negative correlation between Cho/Naa in the left entire CST and forced vital capacity, negative correlation between Cho/Naa in the left CST and right and left finger tap rate, negative correlation between Cho/Naa in left MC and semiovale centrum and right finger tape or forced vital capacity
Han et al., [85]	15/15	NAA/Cr peak was low in the MC and PLIC, Glu/Cr and Glu + Gln/Cr peaks were low in the MC and PLIC	Negative correlation between Glu + Gln/Cr with Norris score
Kalra et al., [88, 89]	63/18	NAA/Cho and NAA/Cr was low in the MC, and Cho/Cr was elevated in the MC	Relation between decreased Naa/Cho in the MC and reduced survival.
Kalra et al., [88, 89]	17/15	NAA/Ins, NAA/Cr and NAA/Cho were low in the MC, and Ins/Cr was elevated in the MC	Negative (p ≤ 0.05)
Liu et al., [87]	19/13	NAA/Cr was low in the MC	Negative
Lombardo et al., [79]	32/19	NAA/Cr was low and ins/Cr and Cho/Cr were elevated in the MC.	Abnormalities were correlated with the El Escorial score
Pohl et al., [91]	70/48	NAA, Pcr + Cr, NAA/Cho and NAA/(Pcr + Cr) were low in the MC. At 12 months, NAA/Cho was low and Cho/(Pcr + Cr) was elevated	Not available
Pyra et al., [59]	14/14	NAA/Cho and NAA/Cr were low in the MC and corona radiata, and Cho/Cr was elevated in the MC	Correlation between Naa/Cho peak in MC and DD, negative correlation between Naa/Cho in MC and corona radiata and DPR
Rooney et al., [86]	10/9	NAA/(Cho + Cr) was low in the MC and CST but not in other regions	Correlation between Naa/(Cho + Cr) in the MC and maximum finger tape rate
Stagg et al., [47]	13/14	NAA was low along the CSTs	Correlation between Naa peak along CSTs and ALSFRS-R
Unrath et al., [96]	8/0	Progressive decrease over time in the NAA peak throughout the MC	Progressive decrease of Naa/(Cr + Cho) in the less affected hemisphere. Correlation between Naa and the more or less affected side (ALSFRS subscore). Correlation between Naa/(Cr + Cho) and the less affected side (ALSFRS subscore)
Verma et al., [93]	21/10	NAA/Cho was low in the right lingual gyrus, parts of the occipital lobe, left supramarginal gyrus and left caudate. NAA/Cr was low in the right MC, left frontal inferior operculum, right cuneus, parts of the occipital lobe, left caudate and left Heschl gyrus	Not available

*MC* motor cortex, *CST* corticospinal tract, *PLIC* posterior limb of the internal capsule, *WM* white matter, *NAA* N-acetylaspartate, *Cho* choline, *Gln* glutamine, *Glu* glutamate, *PCr* phosphocreatine, *Cr* creatine or creatine + Pcr (the distinction is not relevant for interpretation), *DD* disease duration, *DPR* disease progression rate

MRS is a non-invasive means of assessing brain metabolism, based on the chemical properties of molecular structures. Results are expressed as peaks. The main brain metabolites monitored with MRS include N-acetyl-aspartate (NAA), choline (Cho, a cell membrane marker), creatine (Cr) and phosphocreatine (Pcr). The Cr + Pcr peak is a marker of energy metabolism; the level of Cr is assumed to be stable and is used to calculate the metabolite ratio. At a field strength of 1.5 T, the peaks for glutamine (Gln), glutamate (Glu, an excitatory neurotransmitter) and gamma aminobutyric acid (GABA, an inhibitory neurotransmitter) overlap to form a complex referred to as “Glx”; higher field strengths are required for more accurate quantification of this metabolites. The simple sugar myo-inositol (Ins) is absent from neurons but present in glial cells. Hence, elevated brain levels of Ins are associated with glial proliferation, whereas low levels are associated with glial destruction [82–84].

The main results for the CST concern the ratio between the NAA peak on one hand and the Cho and Cr + Pcr peaks on the other. In ALS patients, low levels of NAA have been observed in the precentral corticosubcortical region [85–91], in the PLIC [85] and in entire CSTs [55, 59, 86, 92]. Glu and Glu + Gln were found to be elevated in the precentral corticosubcortical region and in the PLIC [85], whereas Cho was found to be elevated in the precentral region [88, 93, 94] and along the CSTs [47, 59, 92]. Levels of the inhibitory neurotransmitter GABA were low in the precentral cortex [55, 95], and levels of Ins were low in the precentral corticosubcortical region [89].

Whereas the above-mentioned studies used defined voxels for MRS acquisition, whole-brain spectroscopy has revealed low NAA/Cho or NAA/Cr ratios in many other regions [93].

Follow-up studies have shown a decrease in the NAA peak in the precentral cortex (with no differences for other metabolites) [96], a decrease in the NAA/Cho peak and an increase in the Cho/Cr peak [91].

##### Clinical correlations

Patients with definite or probable ALS (according to the El Escorial criteria) had low levels of NAA and high levels of Ins and Cho in the precentral cortex. In patients with possible or suspected ALS, only Cho and (on the left side) Ins were elevated. In another study of suspected ALS patients, only right-side Ins and right-side Cho were elevated [79]. Low NAA and high Cho and Ins levels are associated with clinical disease severity [94]. Lower NAA levels in the precentral gyrus, disease duration and disease progression were intercorrelated [59], and a low NAA/Cho ratio was associated with poor survival [88].

##### Diagnostic accuracy

In a recent study, the combination of MRS and DTI yielded a sensitivity of 0.93 and a specificity of 0.85, whereas the DTI data alone gave values of 0.86 and 0.70, respectively [55]. A criterion combining the MRS data, hypointensity in the precentral gyri and hyperintensity in the CSTs yielded a sensitivity of 0.78 and a specificity of 0.82; when each of the three parameters was considered alone, the sensitivity and specificity values did not exceed 0.71 and 0.75, respectively [90].

#### Iron imaging

Iron overload appears to be involved in the physiopathology of ALS [8, 97]. However, very few studies have analysed the iron content in the brain of ALS patients. A variety of techniques are available, including relaxometry (T2, T2* or R2*) and the susceptibility-weighted imaging [98, 99] (SWI, which is probably more sensitive than relaxometry [100]). Multi-echo T2* and R2* sequences are sensitive to magnetic field inhomogeneities. The value of R2* is measured by voxel-by-voxel modelling of the exponential decrease in signal [101, 102]. In post-mortem studies, R2* values have been found to be correlated with the iron content [103].

#### Susceptibility-weighted imaging and quantitative susceptibility mapping (QSM)

SWI is derived from T2* sequences and can be described as a flow-compensated, high-spatial-resolution T2* weighted sequence. The main advantage over conventional T2* sequence is better contrast [101]. QSM is based on the analysis of phase information. The main sources of phase shifts in biological tissues are the iron, calcium, lipid and myelin contents [104]. The value of QSM in ALS was confirmed in a post-mortem study, in which the iron content of grey matter was assessed more accurately than that of white matter [105].

The initial studies in the field noted a shorter T2 time in the motor cortex of most ALS patients (vs. controls) [106], although this was not confirmed a few years later [107]. Later studies found a correlation being T2* shortening and iron deposition in the microglia of the motor cortex (according to a post-mortem examination) [7, 106, 108]. Iron overload in the motor cortex was also described in SWI studies [109] and qualitative studies [100]; in the later study, there were no differences between ALS patients and controls in terms of T2 or T2*. A few quantitative studies have looked at white matter or deep grey matter [109, 110]. There was a trend towards iron accumulation in CST white matter in a relaxometry study [110]. Iron overload was also present in the red nucleus, substantia nigra, globus pallidus, putamen [109] and caudate nucleus [110]. The use of SWI sequences revealed widespread iron overload and myelin defects in the white matter of all brain lobes and corpus callosum [111]. A retrospective study of iron deposition in motor cortex found that quantitative susceptibility mapping was more accurate than T2*, T2 or FLAIR sequences [112].

A follow-up study has highlighted the progression of a T2 hypointense area in the motor cortex after 6 months, which was strongly and negative correlated with the ALSFRS score [108]. Lastly, changes in SWI sequences in the corpus callosum are correlated with the ALSFRS-R score [111].

#### Imaging of the spinal cord

The few studies to have analysed the spinal cord revealed atrophy in the cervical and upper thoracic regions [113–115]. A DTI study reported low FA, elevated RD and a high magnetization transfer ratio in lateral segments of the cervical spinal cord [114]. Atrophy of the cervical and upper thoracic spinal cord progressed over time, whereas the DTI changes were relatively stable [115]. This observation agrees with the results of a longitudinal brain MRI study showing that grey matter defects (but not white matter defects) progressed over time [78]. Sensory pathways were also involved, with a decreased FA and an elevated MD and elevated RD in the posterior part of the cervical spinal cord [115, 116]. MRS of the cervical spinal cord revealed low NAA/Cr + Pcr and NAA/Ins peaks and an elevated Ins/Cr peak [117].

##### Clinical correlations

Cervical and upper thoracic spinal cord atrophy was correlated with upper arm muscle strength, as measured by manual testing [114]. FA in the cervical spinal cord was correlated with the ALSFRS score. The rate of atrophy in a follow-up study was correlated with the rates of changes of an arm ALSFRS-R score and an arm strength score (in a manual test) [115]. The NAA/Cr + Pcr and NAA/Ins peaks were positively correlated with the ALSFRS-R score and forced vital capacity but negatively correlated with disease progression [117].

#### Functional MRI

Brain fMRI is a recent technique based on blood flow and blood-oxygen-level dependent (BOLD) contrast, which in turn are based on the neurovascular consequences of neuronal activation [118]. The paramagnetic properties of deoxyhaemoglobin lead to signal reduction in gradient echo sequences [101]. Brain fMRI sequences are acquired in the resting state or during performance of a motor, auditory, cognitive, or visual task [119–121]. The resting state sequences are quite similar to T2* sequences [101].

In the resting state, functional connectivity is based on fluctuations of the BOLD signal. In general, seven “default” networks can be identified: default mode, executive control, visual salience, sensorimotor, dorsal attention and auditory networks [118, 122]. Functional connectivity between specific brain regions and/or networks and the rest of the brain can then be analysed [118]. Resting-state analysis can also provide information about local activation (by analysing the amplitude of low-frequency fluctuations (ALFF), for example).

##### The resting state

Resting-state studies have evidenced the widespread reorganization of functional connectivity in ALS patients. This reorganization can be observed as either elevated functional connectivity or low functional connectivity. Functional connectivity was found to be low in several parts of the frontal lobe (the right orbitofrontal cortex, the left inferior frontal cortex [123] and especially the motor cortex [124]) but was elevated in the parietal lobe (the left precuneus and the right angular gyrus [123]) and the left frontoparietal network [123]. This agrees with the elevated sensorimotor connectivity of the left sensorimotor cortex with several regions of the right hemisphere (mainly the cingulate cortex, parahippocampal gyrus and cerebellum crus II) [125]. When compared with alterations of the CSTs (according to FA measurements), there were also changes in right sensorimotor connectivity; the connectivity was elevated if the CST was normal in a DTI analysis but was low if the CST was abnormal [125]. A network-by-network analysis revealed low activity in the default mode network [126] and elevated activity in the sensorimotor network (both of which include parts of the frontal lobe [126, 127], although not the same ones). Thalamic connectivity is also elevated [127].

Resting-state activity (as measured by the ALFF) was elevated in the frontal lobe (the left anterior cingulate, right superior frontal cortex [31] left medial frontal gyrus and right inferior frontal gyrus [128]), the temporal lobe (the right parahippocampal cortex and the left inferior temporal cortex) and the occipital lobe (the middle occipital cortex) [31] but low in the right fusiform gyrus, the visual cortex and right postcentral gyrus [128].

The resting-state connectivity in the frontal cortex is correlated with disease progression and duration [128]. The connectivity between the left sensorimotor cortex and the right parahippocampal gyri and fourth cerebellar lobule is correlated with the ALSFRS-R [125]. Connectivity of the dorsal part of the precentral gyri was correlated with hand strength [124]. Activity in the right parahippocampal gyri was correlated with disease progression, whereas activity in the left anterior cingulate and left temporal gyrus was correlated with cognitive parameters [31].

##### Activation in fMRI

In motor tasks, activation of the motor cortex was elevated [129–131] and spread widely across the supplementary motor area, the premotor cortex [132] and the parietal somatosensory cortex [129, 132]. Inhibition was low [130]. Activation of the ipsilateral sensorimotor cortex was possibly linked to a compensatory mechanism in ALS [133]. A visual task was associated with poor activation of the secondary visual cortex and strong activation of associative areas, whereas an auditory task produced delayed activation in the secondary auditory cortex, and somatosensory stimulation produced prolonged activation of the right inferior frontal gyrus and right posterior insular cortex [134]. In a modified “go”/“no-go” task, activation of the primary motor cortex, other motor areas, the frontal lobe, the insula and the cerebellum was found to decrease over a three-month period [135]. Using another type of stimulus, activation of the hippocampus also fell after 3 months [135]. In a theory-of-mind task designed to evaluation the mirror neuron system, ALS patients displayed abnormally strong activation of the right anterior cortical areas [136]. A link between the severity of motor weakness and the pattern of activation has never been observed, although the ALSFRS-R score was correlated with the signal change in the sensorimotor cortex in one study [129]. When considering the clinical phenotype, only a bulbar-onset subgroup showed low activation of the motor cortex during tongue movements [131]. Shifts in cerebellar or hippocampal activation were correlated with the ALSFRS-R score.

## Discussion

When considering the data as a whole, the most disease-sensitive MRI patterns are located in motor regions (and especially along the CST from the cortex to the spinal cord). However, ALS also affects the brain more broadly; other parts of the frontal lobe, the temporal lobe, the hippocampus, the parietal lobe, the cingulum and the insula are commonly involved, whereas the occipital lobe is less frequently involved. For most MRI parameters, patient vs. control differences are mostly apparent in the left (dominant) hemisphere. The various MRI patterns appear to reflect disease severity, progression and duration. With fMRI, abnormally high levels of activation in the motor cortex are probably related to the disease-related loss of inhibition. Excessive activation appears to be associated with compensation for the loss of function caused by neuron loss.

### Limitations of neuroimaging as a biomarker for ALS

The low average number of participants represents the main limitation: very few published studies have featured more than 30 subjects per group (i.e. ALS and control groups). Together with the differences in the proportions of the various patient phenotypes, this explains the high variability of the results - particularly outside the motor cortex and the CSTs [137].

Only a small proportion of morphometric studies included a whole-brain analysis. The proportion is higher for DTI studies and low (only one study) for MRS. The selection of regions of interest may over-emphasize changes in the CSTs and the motor system and under-emphasize changes outside these areas.

Very few studies had a longitudinal design, which is nevertheless required for the full interpretation of abnormalities. The main difficulty in setting up longitudinal studies relates to the rapid disease progression and the appearance of respiratory failure, which prevents patients from lying in the MRI for any length of time. In studies longer than 6 months, the drop-out rate is usually over 50 %.

fMRI studies have highlighted both elevated activity and low activity in several parts of the brain. The main physiopathological explanations relate to a physiological compensation response or elevated activity due to the loss of inhibitory mechanisms [127].

***Spinal cord imaging*** is a rarely applied imaging modality in ALS, despite the pivotal involvement of the anterior horn of the spinal cord in ALS [137]. Spinal cord imaging appears to be most commonly used in studies of multiple sclerosis, since all studies in this disease context found differences (relative to controls) in the cervical spinal cord. To the best of our knowledge, no studies of the whole spinal cord have been published – perhaps as a result of technical obstacles.

### The biomarker concept

Currently, the diagnosis of ALS is based on the revised El Escorial criteria (also known as the Airlie House criteria), which were established in 1998 and published in 2000 [138]. Although diagnosis during the course of the disease has become relatively easy, the onset of ALS can be masked by clinical overlap with many other diseases [139]. The ideal biomarker is not only sensitive and specific (for various disease phenotypes) early in the disease process but must also easy to access [140]. In ALS, the typical time interval of 12 months between the initial symptoms and disease diagnosis makes this challenge tougher still [140–142]. The best way of determining early biomarkers for ALS may be to monitor cohorts of presymptomatic patients identified by genetic studies [143]. Although wet biomarkers based on blood or CSF analysis are relatively accessible, there are many candidates and study-to-study reproducibility appears to be quite low [5, 140].

### Perspectives

There is currently a need for a robust clinical-radiological description of ALS using MRI as a function of the disease progression, the various endophenotypes and left/right asymmetry (in terms of upper and lower motor neuron impairment, bulbar involvement, and the cognitive profile ranging from frontotemporal dementia, apathy and dysexecutive syndrome to mild attention disorders).

Despite the few number of studies, spinal cord could be a promising region of interest, although this approach is currently limited by technical post-processing difficulties and a sizeable number of large scale and longitudinal studies involving the spinal cord is needed to build stronger MRI biomarkers of the spinal cord. It may be of value to combine several techniques (e.g. atrophy mapping and measurements of the iron content in the grey matter). The results of MRI of the spinal cord are well correlated with the clinical impairment and might be a good surrogate marker.

A comprehensive analysis of MRI parameters in very large cohorts of ALS patients might reveal a radiological surrogate marker for use in clinical trials. Several points should be considered for large scale studies: in 2010, the first Neuroimaging Symposium in ALS (NiALS) had the aim of establishing consensus about the various applications of MRI to the study of ALS and the possibility of multicentre collaboration. Consensus criteria for the main MRI sequences (VBM, DTI, fMRI and spectroscopy) and the main clinical dataset were established [144]. The aim of this consensus was to lead large multicentre and longitudinal studies in imaging research or therapeutic trials [144]. <In our days, studies might better assess the most recent discovers in ALS such as genetic parameters (especially C9ORF72 expansion), the knowledge in cognitive and behavioural impairments even if the patients do not fulfil criteria for frontotemporal dementia [145]. There is still a need for longitudinal studies especially in presymptomatic and early phase of the disease [145]. Following this multicentre aim, a very recent study showed in 253 patients and 189 controls from eight international ALS specialised centre more widespread white matter tract change pooling the analyses than in single-centre analysis and reached estimation of neuropathological changes [146].

## Conclusion

The MRI biomarkers appear to be well correlated with disease severity, duration and progression and are more sensitive in the brain and spinal cord motor regions. The built of MRI biomarkers is limited by the clinical various phenotypes but also by the lack of large and longitudinal studies. To date large cohorts with multicentre studies using a standardized MRI protocol are needed.

## Abbreviations

AD: Axial diffusivity
ADC: Apparent diffusion coefficient
ALFF: Amplitude of low frequency fluctuations
ALS: Amyotrophic lateral sclerosis
ALSFRS: Amyotrophic lateral sclerosis functional rating scale
ALSFRS-R: Amyotrophic lateral sclerosis functional rating scale revised
CAFS: Combined assessment of function and survival
Cho: Choline
Cr: Creatine
CSF: Cerebro spinal fluid
CST(s): Cortico-spinal tract(s)
DTI: Diffusion tensor imaging
FA: Fractional anisotropy
fMRI: Functional magnetic resonance imaging
GABA: Gamma aminobutyric acid
Gln: Glutamine
Glu: Glutamate
Glx: Glutamine and glutamate
Ins: myo-inositol
MD: Mean diffusivity
MRI: Magnetic resonance imaging
MRS: Magnetic resonance spectroscopy
NAA: N-acetyl-aspartate
NiALS: Neurimaging symposium in amyotrophic lateral sclerosis
Pcr: Phosphocreatine
PD: Parkinson’s disease
PLIC: Posterior limb of internal capsule
QSM: Quantitative susceptibility mapping
RD: Radial diffusivity
SWI: Susceptibility weighted imaging
VBM: Voxel-based morphometry
